# How likely are Eastern European and central Asian countries to achieve global NCD targets: multi-country analysis

**DOI:** 10.1186/s12889-024-20186-5

**Published:** 2024-10-05

**Authors:** Anastasiya Dumcheva, Jaakko Nevalainen, Tiina Laatikainen, Pekka Nuorti

**Affiliations:** 1 Tampere University, Kalevantie 4, Tampere, 33100 Finland; 2 University of Eastern Finland, Yliopistonranta 8, 70210 Kuopio, Finland

**Keywords:** Noncommunicable, Eastern Europe, Central Asia, Cardiovascular, Cancer, Diabetes, Chronic respiratory

## Abstract

**Background:**

In Europe, mortality rates from noncommunicable diseases (NCDs) among persons 30–69 years of age (“NCD premature mortality rates”) have declined significantly, except in twelve countries of Eastern Europe and Central Asia, namely Armenia, Azerbaijan, Belarus, Georgia, Kazakhstan, Kyrgyzstan, Moldova, Russia, Tajikistan, Turkmenistan, Ukraine and Uzbekistan. Data on long-term trends in NCD mortality in these countries are limited. We analyzed NCD premature mortality rates, identified change points in NCD mortality trends and forecasted how likely countries are to achieve the global NCD targets, stratified by gender and NCD type.

**Methods:**

We used the 1990–2019 Global Burden of Disease database to analyze NCD trends and identified country-specific change points by using piecewise linear regression. We assessed the likelihood of achieving the global targets for reducing NCD premature mortality rates among persons 30–69 years of age from four NCDs: cancers, diabetes, cardiovascular and chronic respiratory diseases. The global NCD targets are 25% reduction in mortality from 2010 to 2025 (WHO 25X25 target) and 33%—from 2015 to 2030 (SDG 3.4.1). We applied the analysis to both genders and four NCDs.

**Results:**

Only Kazakhstan and Russia are likely to achieve the global NCD targets. For Kazakhstan, WHO 25X25 and SDG 3.4.1 global targets for mortality rates were 494.3 and 374.8 per 100,000 population respectively; the corresponding predicted values (PVs) were 360.6 [CI 260.1–461.1] and 245.1 [CI 113.4–376.8]. For Russia, WHO 25X25 and SDG 3.4.1 global targets were 560.5 and 442.8 per 100,000 population respectively; the corresponding PVs were 427.7 [CI 270.3–585.1] and 311.0 [CI 102.8–519.1]. Achieving NCD global targets is less likely for Kyrgyzstan, while it is unlikely for the rest of countries. Most countries had higher mortality rates and slower progress among men compared with women. The likelihood of achieving overall global NCD targets was mainly explained by reduction in cardiovascular mortality.

**Conclusions:**

In most Eastern Europe and Central Asia countries, progress towards achieving NCD global targets is slow, or there’s a reverse trend. Further quantitative and qualitative research is needed to understand the underlying reasons. Separate indicators are needed to monitor trends for cancers, diabetes and chronic respiratory diseases.

**Supplementary Information:**

The online version contains supplementary material available at 10.1186/s12889-024-20186-5.

## Background

Noncommunicable diseases (NCDs), including cardiovascular diseases, cancer, chronic respiratory diseases, diabetes mellitus and mental health disorders threaten health, economic and social development in all countries around the globe [1]. In 2019, the NCDs accounted for around 74% of all deaths and 60% of disability-adjusted life years in the world [2], leading to a loss of 75% of global Gross Domestic Product in 2010 (US$ 63 trillion) [3]. NCDs share five common risk factors: tobacco use, unhealthy diet, physical inactivity, alcohol use, and air pollution [4].

NCDs have no sufficient or necessary single cause, and the same risk factors can lead to the development of various NCDs. Moreover, different NCDs often occur together and one NCD may affect the development of other NCDs [5, 6]. In addition, other diseases, such as infectious diseases may be associated with the development of certain NCDs [6]. Limited access to essential health services also contributes to NCD occurrence [7]. The multi-factorial etiology of NCDs presents substantial challenge for countries to develop and implement effective prevention and control strategies at the population and individual level.

The World Health Organization (WHO) has developed a list of the most cost-effective population- and individual-level intersectoral interventions to tackle NCDs; so called “Best Buys” [8]. The evidence-base for effective actions is solid, but progress towards reducing NCDs differs significantly between countries. Low and middle-income countries have made slowest progress in decreasing NCD premature mortality rates [1].

In Europe, NCD premature mortality rates among persons 30–69 years of age have declined significantly in the past decade, except in twelve countries of Eastern Europe and Central Asia, where slow declining trend has been documented [9]. These countries are Armenia, Azerbaijan, Belarus, Georgia, Kazakhstan, Kyrgyzstan, Moldova, Russia, Tajikistan, Turkmenistan, Ukraine and Uzbekistan. Estimates suggest that it will take about 50 years for these countries to achieve NCD premature mortality levels of countries that joined the European Union before May 2004 if the current trends in NCDs mortality continue [9].

Overall NCD progress is commonly monitored with two global NCD targets, such as 1) WHO 25X25 target: a 25% relative reduction in the premature mortality from cardiovascular diseases, cancers, diabetes mellitus, and chronic respiratory diseases (The four major NCDs) among persons 30–69 by 2025 compared with 2010 level (WHO 25X25 global target or global NCD target 2025) [10] and 2) one-third relative reduction in the premature mortality from four major NCDs among persons 30–69 by 2030, compared with 2015 level (The Sustainable Development Goal (SDG) 3.4.1 or global NCD target 2030) [11, 12].

The purpose of this research was to 1) analyze trends of the premature mortality rates from four major NCDs over 1990–2019 among persons 30–69 years of age in twelve countries (Armenia, Azerbaijan, Belarus, Georgia, Kazakhstan, Kyrgyzstan, Moldova, Russia, Tajikistan, Turkmenistan, Ukraine and Uzbekistan); 2) identify change points in those trends; 3) assess whether the trends differ by gender and four major NCDs; and 4) assess the likelihood of achieving global NCD target 2025 and 2030, stratified by gender and type of NCD.

## Methods

We used the Global Burden of Disease, Injuries, and Risk Factors Study (GBD) database from 1990 to 2019 [13], which allowed using long term trend data in estimating the likelihood of achieving global NCD targets. GBD is the most comprehensive free-access global database of health estimates on mortality, morbidity and disability and risk factors associated with various diseases. Importantly, GBD provides health estimates by age, sex, country and larger geographical regions over the last four decades. The GBD is led by the Institute for Health Metrics and Evaluation at the University of Washington, U.S.A and is supported by research collaborators around the globe [14]. The primary data sources for producing health estimates include administrative data from various governmental registries, census data, demographic and epidemiologic surveillance, environmental monitoring, population surveys and scientific literature [15]. As such, GBD provides comprehensive data from available sources allowing for comparability between countries and geographical regions.

We selected GBD categories that provided the best matches with the ICD-10 codes as they are defined by SDG 3.4.1 for four major NCDs [16] and downloaded data using GBD Result tool [2]. Supplementary Table 1 compares the ICD-10 codes for four major NCDs included in NCD global target definitions with GBD categories. Total number of NCD deaths for the population 30–69 years of age was downloaded by using 5-year age groups (30–34, 35–39, 40–44, 45–49, 50–54, 55–59, 60–64, 65–69), summed up and divided by the total population of the same age categories provided by GBD and presented as mortality rates per 100,000 population per year. We assumed that global NCD targets are applicable to overall NCD premature mortality rates, as well as to both genders and four major NCDs. Separate analyses were conducted for men, women, and four major NCDs.

As the first step, we performed a simple linear regression analysis to assess the changes in NCD premature mortality rates over 1990–2019 for overall NCD trends, and by gender and four major NCDs. Because the trends were not consistent over the whole period, we extended this to a piecewise linear regression model (also known as segmented linear regression) [17], in order to capture different trends in various time periods for the overall NCDs premature mortality, gender and four major NCDs. We chose candidate change points first by visual inspection of the NCD premature mortality rates in each country over 1990–2019. Each change point was then included into the model such that a level change as well as a change in the slope of the trend were allowed. We did not consider change points closer to each other than three years, because shorter periods than that would not reflect longer term trends within the countries. The final change points were determined by the minimization of the Akaike Information Criterion (AIC) [18]. The model also included an autocorrelation component. A maximum of two change points was usually enough to achieve sufficient fit (defined as a multiple R2 > 0.8). Only in countries where this level was not achieved, three change points were used. The place (year) and number of change points was sometimes different for the overall trend of NCD premature mortality and the one for men, women, cardiovascular diseases, cancers, diabetes, or chronic respiratory diseases.

The best-fitting model was further used for making predictions for how likely the countries are to achieve the overall NCD targets by 2025 and 2030, as well as applied similar targets by gender and four major NCDs types. This included calculating predictions of NCD rates and their confidence intervals (CIs) for 2025 and 2030 years (Supplementary Table 2). Finally, we made a calculation of the global NCD targets’ levels for 2025 and 2030 in line with the global NCD targets’ definitions and compared those with their predicted values and CIs.

We conducted a sensitivity analysis on NCD premature mortality rates with simple linear regression using shorter time periods to investigate how robust our predictions were. Specifically, for predicting the likelihood to achieve NCD target 2025 we used 2010–2019 time period, and for NCD 2030 target – 2015–2019 one. We further compared the results of both methods to support data interpretation.

The data was analyzed using R software program, version 4.3.1 [19, 20].

## Results

### Overall NCD trends

The location of the final change points in piecewise linear regression allowed identifying and distinguishing several time periods (referred as “Periods”) with consistent trends of NCD premature mortality rates in the countries. The trends were varied between Periods indirection (upward trend that changed to downward one, or vice versa), or steepness of the slope (a slower or faster change). Occasionally, we found a level change in NCD premature mortality rates in between identified Periods. For each country, the Periods were numbered sequentially. In total, we identified three Periods in the trend of overall NCD premature mortality rates for most countries and four Periods in Moldova and Ukraine.

**Fig. 1:**
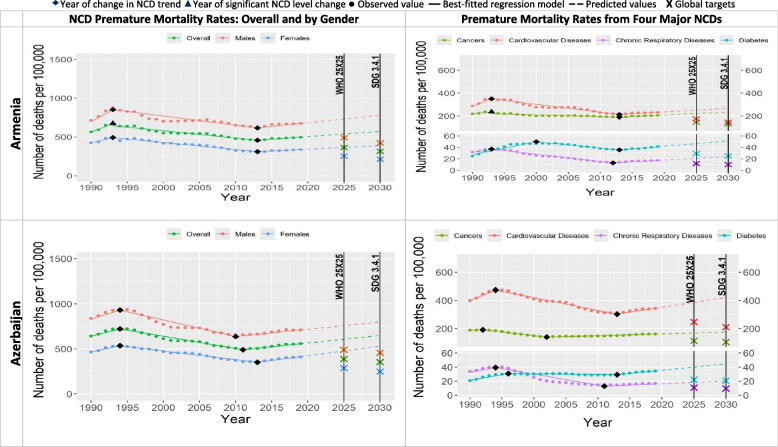
NCD premature mortality rates from cardiovascular disease, cancers, chronic respiratory diseases and diabetes mellitus in Armenia and Azerbaijan among 30–69 years old during 1990–2019 and the likelihood of achieving Global NCD Targets

**Fig. 2:**
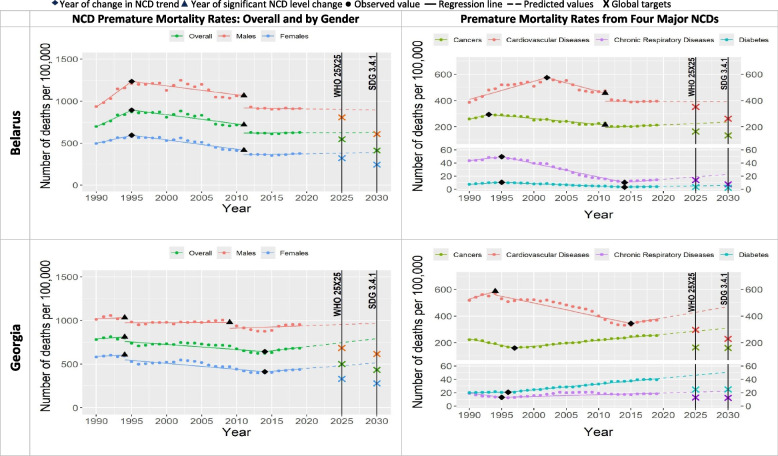
NCD premature mortality rates from cardiovascular disease, cancers, chronic respiratory diseases and diabetes mellitus in Belarus and Georgia among 30-69 years old during 1990-2019 and the likelihood of achieving Global NCD Targets

**Fig. 3:**
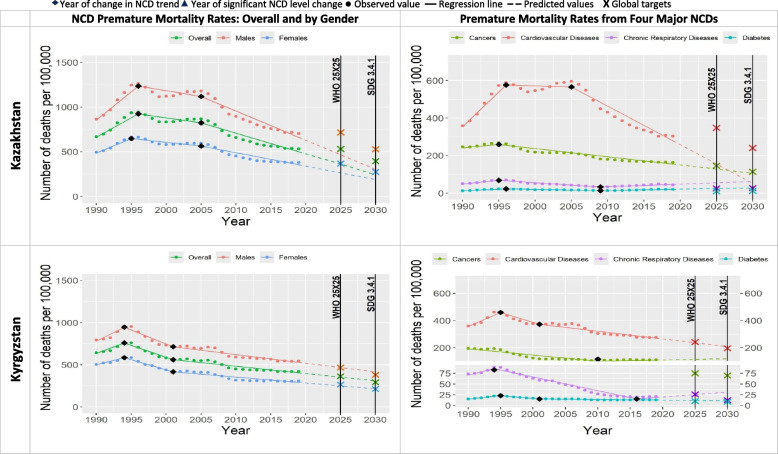
NCD premature mortality rates from cardiovascular disease, cancers, chronic respiratory diseases and diabetes mellitus in Kazakhstan and Kyrgyzstan among 30-69 years old during 1990-2019 and the likelihood of achieving Global NCD Targets

**Fig. 4:**
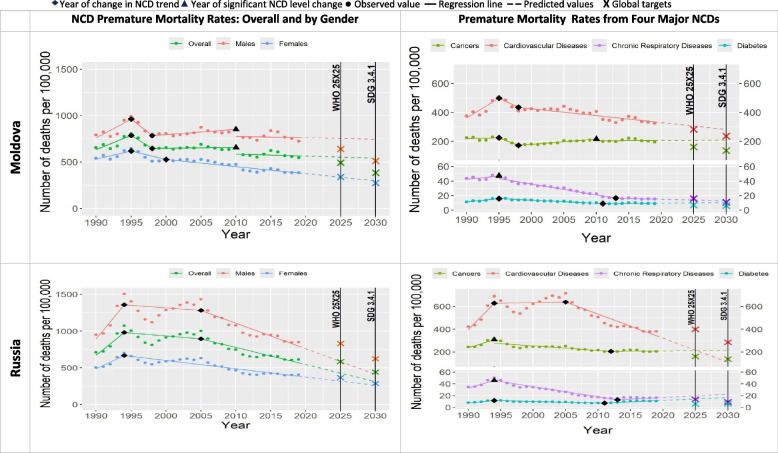
NCD premature mortality rates from cardiovascular disease, cancers, chronic respiratory diseases and diabetes mellitus in Moldova and Russia among 30-69 years old during 1990-2019 and the likelihood of achieving Global NCD Targets

**Fig. 5:**
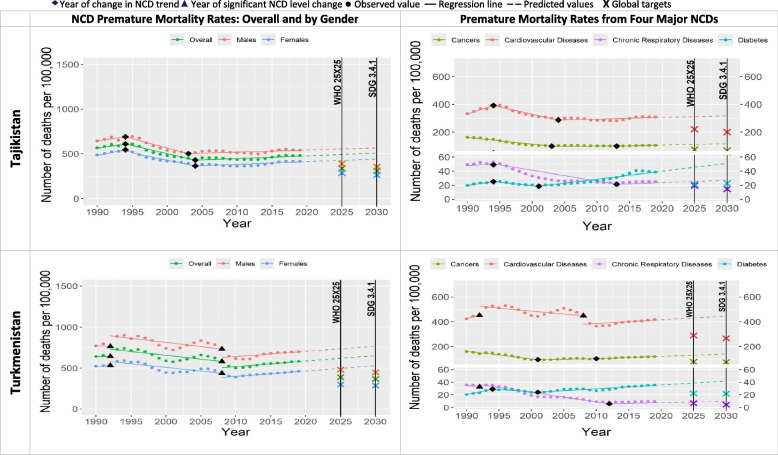
NCD premature mortality rates from cardiovascular disease, cancers, chronic respiratory diseases and diabetes mellitus in Tajikistan and Turkmenistan among 30-69 years old during 1990-2019 and the likelihood of achieving Global NCD Targets

**Fig. 6:**
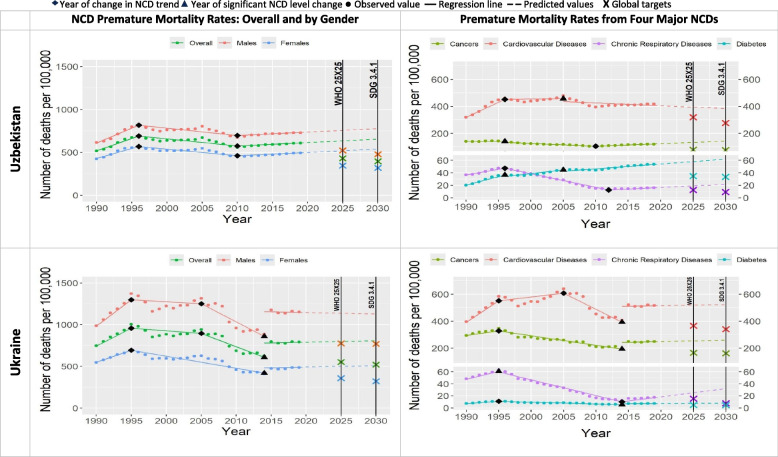
NCD premature mortality rates from cardiovascular disease, cancers, chronic respiratory diseases and diabetes mellitus in Uzbekistan and Ukraine among 30-69 years old during 1990-2019 and the likelihood of achieving Global NCD Targets

Since 1990, NCD premature mortality rates increased an average of 22% in all countries over the next two to six years (Table 1 and Figs. 1, 2, 3, 4, 5, 6). The greatest increases were in Russia, Kazakhstan and Uzbekistan with 46%, 40% and 33% increase respectively (referred to “Period 1”). Period 1 ended in two to six years in all the countries. The NCD premature mortality rates were then followed by a decreasing trends in each Eastern Europe and Central Asia country that led to an average 19% reduction in NCD premature mortality rate in all the countries (referred to “Period 2”). There was more variation in the length of this period, ranging from three to twenty years. “Period 3” for five countries was characterized by new increase in NCD premature mortality rates that continued until 2019, namely for Armenia (since 2013), Azerbaijan (since 2011), Georgia (since 2014), Tajikistan (since 2004) and Uzbekistan (since 2010). For four countries Period 3 was marked with the continued progress at either faster or slower pace compared with Period 2 to further decline the NCD premature mortality rate till 2019, namely for Belarus, Kazakhstan, Kyrgyzstan, and Russia. Turkmenistan experienced almost no change in NCD premature mortality rate from 2008 to 2019. Moldova experienced the increased NCD premature mortality rate from 1998 to 2010 (“Period 3”) followed by decrease from 2010 to 2019 (“Period 4”). Ukraine experienced a stable decrease in NCD premature mortality rates from 2005 till 2014 (“Period 3”) with further sharp increase from 2014 to 2019 (“Period 4”).

**Table 1:** Total NCD premature mortality rates from cardiovascular, cancers, diabetes mellitus, chronic respiratory diseases among 30–69 years old (SDG 3.4 definition) in twelve Eastern Europe and Central Asia countries over 1990–2019—estimates from a piecewise linear regression

	**Period 1**			**Period 2**			**Period 3**			**Period 4**				
	**Year**^**a**^	**NCD mortality**^**b**^	**% change**	**Year**	**NCD mortality**	**% change**	**Year**	**NCD mortality**	**% change**	**Year**	**NCD mortality**	**% change**	**Year**	**NCD mortality**
**Armenia**	**1990**	562.0	+ 19.6	**1993**	672.3	-32.1	**2013**	456.6	+ 8.9				**2019**	497.1
**Azerbaijan**	**1990**	641.0	+ 12.7	**1994**	722.3	-32.0	**2011**	490.9	+ 13.5				**2019**	557.3
**Belarus**	**1990**	698.1	+ 28.1	**1995**	894.5	-19.6	**2011**	719.1	-13.5				**2019**	622.3
**Georgia**	**1990**	788.7	+ 2.8	**1994**	810.6	-20.6	**2014**	643.6	+ 7.2				**2019**	690.2
**Kazakhstan**	**1990**	662.2	+ 39.6	**1996**	924.6	-11.0	**2005**	822.6	-39.3				**2019**	499.2
**Kyrgyzstan**	**1990**	635.4	+ 19.6	**1994**	759.9	-26.2	**2001**	560.8	-27.7				**2019**	405.4
**Moldova**	**1990**	633.9	+ 23.9	**1995**	785.3	-17.8	**1998**	645.7	+ 1.5	**2010**	655.5	-13.7	**2019**	565.4
**Russia**	**1990**	673.0	+ 45.9	**1994**	981.7	-8.9	**2005**	894.7	-36.5				**2019**	567.8
**Tajikistan**	**1990**	561.0	+ 8.6	**1994**	609.1	-29.8	**2004**	427.4	+ 10.6				**2019**	472.7
**Turkmenistan**	**1990**	638.5	+ 0.8	**1992**	643.3	-9.8	**2008**	580.1	-0.3				**2019**	578.1
**Uzbekistan**	**1990**	517.8	+ 33.4	**1996**	690.5	-16.8	**2010**	574.5	+ 6.3				**2019**	610.4
**Ukraine**	**1990**	743.1	+ 28.8	**1995**	957.3	-6.5	**2005**	895.0	-32.0	**2014**	608.4	+ 29.5	**2019**	787.7

Data source: GBD Study 2019

^a^Year for the beginning of the observation period

^b^NCD mortality is presented as number of deaths per 100,000 of the population per year

Table 2 and Figs. 1, 2, 3, 4, 5, 6 show that only two countries are – based on the data until 2019 – likely to achieve both global NCD targets, namely Kazakhstan and Russia. Kyrgyzstan is less likely to achieve both global NCD targets. The rest of Eastern Europe and Central Asia countries are unlikely or highly unlikely to achieve the global NCD targets.

**Table 2:** Predicted overall NCD mortality rates from cardiovascular diseases, cancers, diabetes mellitus and chronic respiratory diseases among 30–69 years old (SDG 3.4 definition) and global NCD targets for twelve Eastern Europe and Central Asian countries

	**Predicted NCD mortality rate [95% CI]**		**NCD Targets and Likelihood to Achieve**^**a**^	
Year	**2025**	**2030**	**2025**	**2030**
Armenia	**537.6** [486.2–589.0]	**571.3** [495.3- 647.4]	**357.7 (Highly unlikely)**	**320.6 (Highly unlikely)**
Azerbaijan	**607.0** [562.9–651.1]	**648.4** [585.8–711.1]	**380.0 (Highly unlikely)**	**351.7 (Highly unlikely)**
Belarus	**624.1** [530.1–718.0]	**625.6** [486.2–764.9]	**536.2 (Unlikely)**	**404.9 (Highly unlikely)**
Georgia	**746.0** [648.4–843.6]	**792.6** [645.7–939.4]	**505.1 (Highly unlikely)**	**420.2 (Highly unlikely)**
Kazakhstan	**360.6** [260.1–461.1]	**245.1** [113.4–376.8]	**494.3 (Likely)**	**374.8 (Likely)**
Kyrgyzstan	**353.6** [306.1–401.1]	**310.4** [249.7–371.2]	**337.3 (Less likely)**	**289.8 (Less likely)**
Moldova	**551.7** [472.6–630.7]	**540.2** [424.3–656.2]	**492.9 (Unlikely)**	**413.7 (Highly unlikely)**
Russia	**427.7** [270.3–585.1]	**311.0** [102.8–519.1]	**560.5 (Likely)**	**442.8 (Likely)**
Tajikistan	**490.8** [451.8–529.9]	**505.9** [454.8–557.0]	**325.3 (Highly unlikely)**	**305.7 (Highly unlikely)**
Turkmenistan	**624.8** [522.3–727.3]	**651.5** [519.7–783.3]	**374.1 (Highly unlikely)**	**370.0 (Highly unlikely)**
Uzbekistan	**634.4** [572.1–696.7]	**654.3** [567.0–741.6]	**417.7 (Highly unlikely)**	**394.0 (Highly unlikely)**
Ukraine	**800.0** [567.8–1032.1]	**807.0** [477.8–1136.1]	**515.4 (Highly unlikely)**	**530.0 (Unlikely)**

^a^Likelihood to achieve is assessed as “Likely” if prediction and more than 75% of CI is below target, “Less likely” if prediction is no more than 25% above the target and included in CI, “Unlikely” if prediction is more than 25% above the target, but included in CI or “Highly unlikely” if CI does rule out the target value

Sensitivity analysis confirmed most of the findings. However, it suggested more optimistic prediction for Belarus and Moldova to achieve global NCD target 2025 moving them from the categories “unlikely” and “highly unlikely” to the category “less likely”. At the same time, the sensitivity analysis provided more pessimistic predictions to achieve global NCD target 2025 for Kyrgyzstan (“highly unlikely”) and to achieve global NCD target 2030 for Russia (“less likely”), as well as to Kazakhstan, Kyrgyzstan and Ukraine (“highly unlikely).

### Trends by gender

In all the countries, NCD premature mortality rates among men were estimated to be markedly higher than among women throughout the observed period (Figs. 1, 2, 3, 4, 5, 6). In 1990, NCD premature mortality rates among men was from 1.3 to 1.9 times higher than among women with the highest, nearly two-fold differences in Belarus, Russia, Azerbaijan, and Ukraine. By 2019, gender gap was still present and further increased, where the NCD premature mortality rates among men was 1.3 to 2.4 times higher than among women being the highest, more than two-fold in Belarus, Ukraine, Georgia, Russia, Armenia and Moldova. On average, in Eastern Europe and Central Asia countries NCD premature mortality rates decreased by 8% among men and by 23% among women by 2019 compared with 1990. Less than 10% reduction of NCD premature mortality rates among men from 1990 to 2019 was observed in Armenia, Georgia, Belarus, and Moldova, whereas NCD premature rates mortality among women in these countries decreased by 25%, 32%, 34%, and 39%, respectively.

Figures 1, 2, 3, 4, 5, 6 show the likelihood to achieve NCD global targets among men and women followed mainly the same patterns as for overall NCD premature mortality rates.

### Trends by NCD type

Premature mortality rates due to cardiovascular diseases was the main contributor to the overall NCD premature mortality and mostly defined the pattern of mortality from four major NCDs (Figs. 1, 2, 3, 4, 5, 6). As such, countries that were likely to reduce cardiovascular diseases premature mortality rates by 2025 and 2030 in line with NCD global target definitions, were also likely to achieve them. Only two countries, Kazakhstan and Russia, were likely to achieve both global NCD targets for cardiovascular diseases, while for Kyrgyzstan it is less likely, but possible. Moldova was “less likely” to achieve the NCD global target 2025, and “highly unlikely” to achieve global target 2030. Armenia, Azerbaijan, Georgia, Tajikistan, Turkmenistan and Ukraine had a clear increasing trend of premature mortality rates from cardiovascular diseases over the final 4 to 11 years of the observed period.

Premature mortality rates due to cancers were the second largest contributor to the overall NCD premature mortality rates. Only Kazakhstan was likely to reduce premature mortality rates from cancers in line with NCD global target definitions. The rest of the countries had increasing premature mortality rates due to cancers over the last 5–22 years of the observed period.

Premature mortality rates due to diabetes and chronic respiratory diseases contributed the least to overall NCD premature mortality due to relatively low rates over the period of 1990–2019. None of the countries were likely to achieve NCD global targets for diabetes, quite the opposite: an increase in premature mortality rates from diabetes was observed everywhere. As for chronic respiratory diseases, most countries were not likely to achieve both NCD global targets with a few exceptions: Moldova, Turkmenistan, and Kyrgyzstan that were likely or less likely to achieve NCD global target 2025 and/or 2030. Premature mortality rates due to chronic respiratory diseases were increasing in Armenia, Azerbaijan, Belarus, Georgia, Kazakhstan, Kyrgyzstan, Tajikistan, Turkmenistan, Uzbekistan, Russia and Ukraine.

## Discussion

The main findings of our study were: First, most of the Eastern Europe and Central Asia countries are unlikely to reduce NCD premature mortality rates in line with NCD global targets definitions for 2025 and 2030. Only two countries are likely to achieve both NCD global targets 2025 and 2030, namely Kazakhstan, and Russia. For Kyrgyzstan, it is less likely to achieve both NCD global targets. The other countries are unlikely or highly unlikely to achieve both NCD global targets 2025 and 2030. Second, major gender disparities exist across Eastern Europe and Central Asia countries with higher NCD premature mortality rates and slower progress in NCD premature mortality rates reduction among men than women. Third, the likelihood of achieving overall global NCD targets was mainly explained by the progress in reducing premature mortality rates due to cardiovascular diseases. For other three NCDs, no country is likely to achieve both NCD global targets for cancers, except Kazakhstan. In all countries the increasing NCD premature mortality rate due to diabetes makes them unlikely to achieve global NCD targets. As for chronic respiratory diseases, most countries are ulikely to achieve both global NCD targets, except for Moldova, Turkmenistan, and Kyrgyzstan which are likely or less likely to achieve global NCD target 2025 and/or 2030.

Findings of our study are consistent with previous research that documenting high levels of NCD premature mortality rates and low likelihood in achieving the NCD SDG 3.4.1 goal in Eastern Europe and Central Asia countries [21–23], especially among men [21, 22]. Further, Martinez et al. [24] similarly reported upward trends and high levels of premature avertable NCD mortality in Ukraine, Turkmenistan and Uzbekistan. Our findings are also consistent with previous research indicating that trends in cardiovascular diseases are the main contributor to whether or not countries reach the global NCD targets [21–23]. Increases in diabetes premature mortality is also well documented [23].

The results of our study provide a less optimistic picture than some previous studies for achieving NCD global target 2030 (SDG 3.4.1) for twelve Eastern Europe and Central Asia countries. For example, Bennett JE et al. [21] estimated that Armenia, Azerbajan, Belarus, Kazakhstan, Moldova, Russia and Ukraine are likely to achieve SDG 3.4.1 for women, and Belarus and Kazakhstan, Russia – for men; whereas our analysis found such likelihood only for Kazakhstan and Russia. This difference may have resulted from the utilization of more recent data (up to 2019) and capturing more recent changes in NCD trends in our study, whereas previous studies used earlier datasets.

Our analysis might still suggest that the scenario for countries in achieving global NCD targets is optimistic, since our data was before the COVID-19 pandemic. The pandemic significantly reduced access to NCD care in Eastern Europe and Central Asia and other countries [25, 26], and recently published GDB estimates suggest that the global all-cause mortality increased from 2020 to 2021 and that the leading cause of death in 2021 was ischemic heart disease, followed by COVID-19, stroke and chronic obstructive pulmonary diseases [27, 28]. While the COVID-19 pandemic shifted the rankings of leading causes of deaths, lowering stroke to the third-leading and chronic obstructive pulmonary disease to the fourth-leading position in 2021 compared with 2019, the highest age-standardised mortality rate in 2021 was due to NCDs [28] Recent GBD estimates indicate that from 1990 to 2021, most countries in Eastern Europe and Central Asia gained years of life expectancy due to reduced mortality from ischemic heart disease, strokes, neoplasms and chronic obstructive pulmonary diseases. However, these gains were offset by increased mortality from diabetes and chronic kidney disease [28].

TOur study period did not include the major social, economic, and humanitarian crisis in the European region caused by the full-scale Russian invasion and ongoing war in Ukraine which led to further disruption of NCD services [29]. These factors potentially have contributed to a further increase in NCD premature mortality rate in Ukraine and, possibly, also in neighboring countries, such as Moldova, Belarus, Georgia, Russia and others. As a result, even fewer countries of Eastern Europe and Central Asia might be on track to achieve the NCD global targets.

Our research has several strengths. First, using the open access GBD database allowed retrieval of reliable, internationally recognized data on NCD premature mortality for all twelve countries of interest. Second, a longer time period from 1990 to 2019 enabled better predictions than the previously used approach of using 2010 or 2015 as the reference years for assessing global NCD targets [21–23]. The sensitivity analysis using shorter time periods confirmed most of the findings; at the same time, predictions for some countries was influenced by NCD premature mortality rates during recent years and might not completely capture the direction and intensity of their developments. Third, applying the piecewise linear regression method allowed us to assess the change points for major changes in trends of NCD premature mortality rates. Further, stratifying NCD targets allowed assessing the likelihood of achieving global NCD targets by gender and four major NCDs different disease groups, not available in previous studies. Finally, our prediction analysis also relied on the implicit assumption of no disruptive events in the countries and such events could compromise the predictions. The fact that our data did not contain the effects of COVID pandemic might give a more realistic forecast of the likelihood of Eastern Europe and Central Asia countries achieving NCD global targets, as the excess mortality rates will likely return to baseline after the pandemic.

This research has also several limitations. First, we recognize that the GBD grouping of ICD-10 codes for four major NCDs does not completely match the ICD codes recommended to be included for SDG 3.4.1 (Supplementary Table 1). While SDG 3.4.1 includes comprehensive ICD codes for cardiovascular diseases (I00-I99), cancers (C00-C97), diabetes mellitus (E10-E14), and chronic respiratory diseases (J30-J98), the groups used by GBD exclude some of these codes. The main differences are for cardiovascular and chronic respiratory disease categories (Supplementary Table 1), where some of the ICD-10 codes associated with premature mortality are excluded (eg. I15-I16 codes related to hypertension; J69, J81 and J86 -related to respiratory diseases). However, many ICD-10 codes that were not included into SDG 3.4.1 definition, are relevant GBD categories, as they include conditions that do not contribute much to mortality rates (eg. B33.2-B33.24, D86.85, G45-46, K75.1, D86, G47.3 codes on rare cardiovascular or chronic respiratory diseases; D00-D49 codes on in-situ or benign neoplasms; Category R codes on several symptoms, signs and abnormal clinical and laboratory findings not elsewhere classified; Category Z codes on factors influencing health status and contact with health services). The above-described differences may have led to underestimation of NCD premature mortality rates in our study mainly due to the exclusion in GBD grouping of several diseases and their ICD-10 codes potentially contributing to the increase in NCD premature mortality rates. Our analysis may therefore be considered as a conservative estimate of NCD premature mortality rates. However, this would not influence our research results for identifying the best-fitted models and prediction estimates to achieve global NCD targets.

Second, our method involved the identification of the position of the change points using model fitting criteria. The implication of this approach was that some predictions were based on shorter trends than others and could be imprecise. However, our sensitivity analyses showed that the main results were robust against alternative choices. Finally, the likelihood of achieving global NCD targets in the prediction models had relatively wide confidence intervals. Assessed countries have undergone several different periods of trends making prediction difficult and including substantial uncertainty.

Our findings suggest several implications for further research. Eastern Europe and Central Asia countries are falling behind in achieving NCD global targets, and many show an upward trend in NCD premature mortality rates in recent years. This calls for comprehensive research in each country as well as further multi-country analysis of Eastern Europe and Central Asia region to understand better the underlying reasons. Also, our study reiterated that the likelihood of achieving NCD global targets is mainly driven by cardiovascular diseases premature mortality rates, emphasizing the importance of effective reduction of mortality from cardiovascular diseases. While making inferences based on mortality related cardiovascular diseases is helpful for SDG progress monitoring at global, regional and national levels, it does not provide sufficient information about the mortality and morbidity from three other NCDs (cancers, chronic respiratory diseases and diabetes) as they are hidden under considerably higher burden of cardiovascular diseases mortality. This suggests that we need separate global indicators for each of the major NCDs to monitor progress towards achieving each of them.

## Conclusions

The findings of our research suggest that further quantitative and qualitative research is needed to understand the underlying reasons for observed developments in NCD premature mortality rates in each country. Finally, the development of separate global indicators is necessary to allow progress monitoring of the premature mortality rates due to cancers, diabetes and chronic respiratory diseases in the countries as they are currently hidden under considerably higher burden of mortality from cardiovascular diseases.

## Supplementary Information

Supplementary Material 1.

Supplementary Material 2.

## Data Availability

We used the GBD 1990-2019 dataset for the current research, which is open and publicly available data source available at https://vizhub.healthdata.org/gbd-results/

## Abbreviations

AIC: Akaike Information Criterion
CI: Confidence interval
COVID-19: Coronavirus Disease 2019/Severe Acute Respiratory Syndrome Coronavirus 2 (SARS-CoV-2)
GBD: Global Burden of Disease, Injuries, and Risk Factors Study
ICD-10: International Statistical Classification of Diseases and Related Health Problems, 10th revision
NCD: Noncommunicable disease
PVs: Predicted values
SDG: Sustainable Development Goal
WHO: World Health Organization
WHO 25X25: WHO target aimed to decrease premature NCD mortality by 25% in 2025
