# Supplementary material for: How likely are Eastern European and central Asian countries to achieve global NCD targets: multi-country analysis
Source: BMC Public Health. 2024 Oct 5;24:2714. doi: 10.1186/s12889-024-20186-5 (PMC11452959; doi:10.1186/s12889-024-20186-5)
Supplement: Supplementary file 1 — Supplementary Material 1. [file 12889_2024_20186_MOESM1_ESM.docx]

**Annex 1. Comparison of ICD-10 Codes Included in SDG 3.4.1 Definition and Relevant GBD Categories**

| **Four major NCDs by SDG 3.4.1 definition** | | **Global Burden of Disease Study 2019** | | | |
| --- | --- | --- | --- | --- | --- |
| **Name of NCD** | **ICD-10 codes included** | **Added GBD categories for analysis** | **ICD-10 codes included in GBD category** | **ICD-10 codes excluded from GBD categories compared to SDG definition:** | **ICD-10 codes included to GBD categories and data anlysis, but not included into SDG definition:** |
| **Cardiovascular disease I15 - Secondary hypertensionIII95I** | **I00-I99** | Cardiovascular diseases  (GBD code 491) | B33.2-B33.24, D86.85, G45-G46.8, I01-I01.9, I02.0, I05-I09.9, I11-I11.2, I11.9, I20-I21.6, I21.9-I27.0, I27.2-I28.9, I30-I38.0, I39-I41.8, I42-I43.8, I44-I44.8, I45-I52.8, I60-I64, I64.1, I65-I83.93, I86-I89.0, I89.9, I95.0-I95.1, I98, I98.8-I99.9, K75.1, R00-R01.2, Z01.3-Z01.31, Z03.4-Z03.5, Z13.6, Z52.7, Z82.3-Z82.49, Z86.7-Z86.79, Z94.1-Z94.3, Z95-Z95.9 | I00 – rheumatic fever without heart involvement | B33.2-B33.24 Viral carditis |
|  |  |  |  | I10 - Essential (primary) hypertension | D86.85 Sarcoid myocarditis |
|  |  |  |  | I15 - Secondary hypertension | G45 Transient cerebral ischemic attacks and related syndromes |
|  |  |  |  | I16 - Hypertensive crisis | G46 Vascular syndromes of brain in cerebrovascular diseases |
|  |  |  |  | I27.1 Kyphoscoliotic heart disease | K75.1 Phlebitis of portal vein |
|  |  |  |  | I5A Non-ischemic myocardial injury (non-traumatic) | R00 Abnormalities of heart beat |
|  |  |  |  | I85 Esophageal varices | R01.2 Cardiac murmurs and other cardiac sounds |
|  |  |  |  | I95.2 Hypotension due to drugs | Z01.3 Encounter for examination of blood pressure |
|  |  |  |  | I95.3 Hypotension of hemodialysis | Z03.4: Observation for suspected myocardial infarction |
|  |  |  |  | I95.8 Other hypotension | Z13.6 Encounter for screening for cardiovascular disorders |
|  |  |  |  | I95.9 Hypotension, unspecified | Z52.7 Heart donor |
|  |  |  |  | I96 Gangrene, not elsewhere classified | Z82.3 Family history of stroke |
|  |  |  |  | I97 Intraoperative and postprocedural complications and disorders of circulatory system, not elsewhere classified | Z82.49 Family history of ischemic heart disease and other diseases of the circulatory system |
|  |  |  |  |  | Z86.7 Personal history of diseases of the circulatory system |
|  |  |  |  |  | Z94.1 Heart transplant status |
|  |  |  |  |  | Z94.2 Lung transplant status |
|  |  |  |  |  | Z94.3 Heart and lungs transplant status |
|  |  |  |  |  | Z95 Presence of cardiac and vascular implants and grafts |
|  |  | Chronic kidney disease due to hypertension  (GBD code 591) | ICD I12-I13.9 |  |  |
| **Cancer** | **C00-C97** | Neoplasms  (GBD code 410) | C00-C07, C08-C19.0, C20, C21-C21.8, C22-C22.4, C22.7-C23, C24-C26.1, C26.8-C26.9, C30-C30.1, C31-C33, C34-C34.92, C37-C37.0, C38-C39.9, C40-C41.4, C41.8-C41.9, C43-C45.2, C45.7, C45.9, C47-C4A, C50-C50.629, C50.8-C52, C53-C54.3, C54.8-C56.2, C56.9-C58.0, C60-C64.2, C64.9-C69.92, C70-C70.1, C70.9-C73, C74-C75.5, C75.8-C79.9, C80-C81.49, C81.7-C81.79, C81.9-C85.29, C85.7-C86.6, C88-C90.32, C91-C93.7, C93.9-C95.2, C95.7-C97.9, D00-D24.9, D26.0-D39.9, D4-D49.9, E34.0, K51.4-K51.419, K62.0-K62.3, K63.5, N60-N60.99, N84.0-N84.1, N87-N87.9, Z03.1, Z08-Z09.9, Z12-Z12.9, Z80-Z80.9, Z85-Z85.9, Z86.0-Z86.03 | C46 Kaposi's sarcoma | D00-D09 In situ neoplasms |
|  |  |  |  |  | D10-D36 Benign neoplasms, except benign neuroendocrine tumors |
|  |  |  |  |  | D37-D49 Neoplasms of uncertain behavior, polycythemia vera and myelodysplastic syndromes |
|  |  |  |  |  | E34.0 Carcinoid syndrome |
|  |  |  |  |  | K51.4 Inflammatory polyps of colon |
|  |  |  |  |  | K62 Other diseases of anus and rectum |
|  |  |  |  |  | K63 Other diseases of intestine |
|  |  |  |  |  | N60 Benign mammary dysplasia |
|  |  |  |  |  | N84.0 Polyp of corpus uteri |
|  |  |  |  |  | N87 Dysplasia of cervix uteri |
|  |  |  |  |  | Z03 Encounter for medical observation for suspected diseases and conditions ruled out |
|  |  |  |  |  | Z08 Encounter for follow-up examination after completed treatment for malignant neoplasm |
|  |  |  |  |  | Z09  Encounter for follow-up examination after completed treatment for conditions other than malignant neoplasm |
|  |  |  |  |  | Z12 Encounter for screening for malignant neoplasms |
|  |  |  |  |  | Z80 Family history of primary malignant neoplasm |
|  |  |  |  |  | Z85 Personal history of malignant neoplasm |
|  |  |  |  |  | Z86 Personal history of certain other diseases |
| **Diabetes** | **E10-E14** | Diabetes mellitus  (GBD code 587) | E08-E08.11, E08.3-E08.9, E10-E10.11, E10.3-E11.1, E11.3-E12.1, E12.3-E13.11, E13.3-E14.1, E14.3-E14.9, R73-R73.9, Z13.1, Z83.3 |  | E08: Diabetes mellitus due to underlying condition |
|  |  |  |  |  | R73.0-R73.9 Elevated blood glucose level |
|  |  |  |  |  | Z13.1: Encounter for screening for diabetes mellitus |
|  |  |  |  |  | Z83.3 Family history of diabetes mellitus |
|  |  | Chronic kidney disease due to diabetes mellitus type 1  (GBD code 997) | E10.2-E10.29 |  |  |
|  |  | Chronic kidney disease due to diabetes mellitus type 2 (GBD code 998) | E11.2-E11.29 |  |  |
| **Chronic respiratory disease** | **J30-J98** | Chronic respiratory diseases  (GBD code 508) | D86-D86.2, D86.9, G47.3-G47.39, J30-J35.9, J37-J39.9, J41-J42.4, J43-J46.0, J47-J47.9, J60-J68.9, J70.8-J70.9, J80-J80.9, J82, J84-J84.9, J90-J90.0, J91, J91.8-J93.12, J93.8-J94.9, J96-J96.92, J98-J99.8, R05.0-R06.9, R09-R09.89, R84-R84.9, R91-R91.8, Z82.5 | J36: Peritonsillar abscess | D86 Sarcoidosis:  D86.1 Sarcoidosis of lymph nodes  D86.2 Sarcoidosis of lung with sarcoidosis of lymph nodes  D86.9 Sarcoidosis, unspecified |
|  |  |  |  | J40 Bronchitis, not specified as acute or chronic | G47.3 Sleep apnea |
|  |  |  |  | J69 Pneumonitis due to solids and liquids | R05 Cough |
|  |  |  |  | J81 Pulmonary edema | R06 Abnormalities of breathing |
|  |  |  |  | J85 Abscess of lung and mediastinum | R09 Other symptoms and signs involving the circulatory and respiratory system |
|  |  |  |  | J86 Pyothorax | R84 Abnormal findings in specimens from respiratory organs and thorax |
|  |  |  |  |  | R91 Abnormal findings on diagnostic imaging of lung |
|  |  |  |  |  | Z82.5 Family history of asthma and other chronic lower respiratory diseases |
