# Supplementary material for: How likely are Eastern European and central Asian countries to achieve global NCD targets: multi-country analysis
Source: BMC Public Health. 2024 Oct 5;24:2714. doi: 10.1186/s12889-024-20186-5 (PMC11452959; doi:10.1186/s12889-024-20186-5)
Supplement: Supplementary file 2 — Supplementary Material 2. [file 12889_2024_20186_MOESM2_ESM.docx]

**Annex 2. Predictions and Target values for NCD Global Targets 2025 and 2030**

| **Eastern Europe and Central Asia Countries & Reference Territories** | **NCD Mortality: Overall, by gender and NCD type** | **Predicted** | | | | | | **NCD Global Targets** | |
| --- | --- | --- | --- | --- | --- | --- | --- | --- | --- |
|  |  | **2025** | CI lower limit | CI upper limit | **2030** | CI lower limit | CI upper limit | **2025** | **2030** |
| **Armenia** | **Total** | **537.6** | 486.2 | 589.0 | **571.3** | 495.3 | 647.4 | **357.7** | **320.6** |
|  | **Males** | **731.3** | 630.6 | 832.1 | **779.3** | 632.6 | 926.1 | **488.5** | **440.4** |
|  | **Females** | **363.9** | 328.0 | 399.7 | **386.4** | 333.4 | 439.3 | **244.1** | **216.3** |
|  | **Cancers** | **222.0** | 206.0 | 238.0 | **235.3** | 211.6 | 259.0 | **146.0** | **133.4** |
|  | **CVD** | **250.4** | 209.6 | 291.2 | **265.9** | 205.8 | 326.1 | **172.3** | **151.3** |
|  | **CRD** | **20.6** | 16.7 | 24.6 | **23.6** | 18.0 | 29.3 | **11.0** | **10.6** |
|  | **DM** | **46.6** | 39.2 | 53.9 | **51.2** | 40.2 | 62.1 | **28.4** | **25.3** |
| **Azerbaijan** | **Total** | **607.0** | 562.9 | 651.1 | **648.4** | 585.8 | 711.1 | **380.0** | **351.7** |
|  | **Males** | **758.4** | 686.3 | 830.5 | **798.6** | 698.9 | 898.3 | **490.3** | **457.5** |
|  | **Females** | **477.9** | 448.0 | 507.7 | **530.6** | 486.5 | 574.7 | **277.5** | **252.4** |
|  | **Cancers** | **168.4** | 162.6 | 174.2 | **174.6** | 167.1 | 182.0 | **110.0** | **103.4** |
|  | **CVD** | **386.9** | 354.5 | 419.4 | **421.8** | 373.8 | 469.8 | **237.7** | **216.8** |
|  | **CRD** | **18.3** | 10.5 | 26.1 | **20.2** | 9.7 | 30.7 | **11.0** | **10.5** |
|  | **DM** | **40.1** | 36.8 | 43.4 | **44.6** | 39.7 | 49.5 | **21.4** | **21.1** |
| **Belarus** | **Total** | **624.1** | 530.1 | 718.0 | **625.6** | 486.2 | 764.9 | **536.2** | **404.9** |
|  | **Males** | **902.0** | 770.8 | 1033.1 | **895.7** | 700.4 | 1090.9 | **797.0** | **601.2** |
|  | **Females** | **380.6** | 316.5 | 444.7 | **387.7** | 293.2 | 482.3 | **309.0** | **234.2** |
|  | **Cancers** | **226.4** | 201.9 | 250.8 | **237.4** | 201.0 | 273.7 | **169.3** | **135.1** |
|  | **CVD** | **392.3** | 317.1 | 467.5 | **391.4** | 280.3 | 502.5 | **350.9** | **259.1** |
|  | **CRD** | **18.9** | 11.6 | 26.3 | **22.8** | 11.7 | 34.0 | **12.4** | **8.4** |
|  | **DM** | **5.2** | 4.0 | 6.3 | **6.0** | 4.3 | 7.7 | **3.5** | **2.4** |
| **Georgia** | **Total** | **746.0** | 648.4 | 843.6 | **792.6** | 645.7 | 939.4 | **505.1** | **420.2** |
|  | **Males** | **954.0** | 883.6 | 1024.4 | **967.8** | 867.3 | 1068.3 | **703.6** | **590.6** |
|  | **Females** | **482.2** | 390.6 | 573.7 | **513.9** | 377.1 | 650.8 | **331.4** | **269.1** |
|  | **Cancers** | **287.0** | 281.4 | 292.6 | **309.5** | 302.5 | 316.6 | **165.3** | **163.5** |
|  | **CVD** | **429.2** | 332.6 | 525.9 | **471.1** | 324.0 | 618.3 | **300.7** | **220.1** |
|  | **CRD** | **21.0** | 16.1 | 25.9 | **22.3** | 16.5 | 28.1 | **14.4** | **11.5** |
|  | **DM** | **46.3** | 45.1 | 47.6 | **50.7** | 49.2 | 52.3 | **24.7** | **25.0** |
| **Kazakhstan** | **Total** | **360.6** | 260.1 | 461.1 | **245.1** | 113.4 | 376.8 | **494.3** | **374.8** |
|  | **Males** | **464.6** | 334.7 | 594.4 | **301.0** | 130.4 | 471.6 | **671.5** | **505.1** |
|  | **Females** | **264.0** | 194.0 | 334.0 | **189.2** | 98.0 | 280.4 | **337.8** | **258.6** |
|  | **Cancers** | **128.1** | 103.9 | 152.3 | **106.3** | 77.3 | 135.4 | **134.7** | **110.9** |
|  | **CVD** | **155.3** | 85.4 | 225.2 | **52.6** | -38.9 | 144.2 | **322.9** | **223.4** |
|  | **CRD** | **55.2** | 47.3 | 63.0 | **62.0** | 51.2 | 72.9 | **26.1** | **28.7** |
|  | **DM** | **24.7** | 21.8 | 27.7 | **28.3** | 24.1 | 32.4 | **10.6** | **11.8** |
| **Kyrgyzstan** | **Total** | **353.6** | 306.1 | 401.1 | **310.4** | 249.7 | 371.2 | **337.3** | **289.8** |
|  | **Males** | **467.4** | 411.8 | 523.0 | **415.9** | 344.6 | 487.2 | **443.3** | **376.6** |
|  | **Females** | **250.0** | 206.6 | 293.4 | **215.3** | 160.0 | 270.6 | **238.6** | **209.5** |
|  | **Cancers** | **113.9** | 82.5 | 145.2 | **118.4** | 75.9 | 160.9 | **78.8** | **74.6** |
|  | **CVD** | **236.8** | 200.6 | 273.0 | **208.5** | 162.3 | 254.8 | **228.7** | **192.8** |
|  | **CRD** | **25.3** | 7.5 | 43.1 | **30.8** | 2.6 | 59.0 | **20.2** | **13.7** |
|  | **DM** | **11.7** | 10.4 | 13.0 | **11.0** | 9.2 | 12.7 | **9.6** | **8.7** |
| **Moldova** | **Total** | **551.7** | 472.6 | 630.7 | **540.2** | 424.3 | 656.2 | **492.9** | **413.7** |
|  | **Males** | **752.7** | 639.8 | 865.6 | **745.4** | 579.8 | 910.9 | **649.0** | **557.5** |
|  | **Females** | **335.6** | 286.2 | 385.0 | **297.5** | 234.4 | 360.6 | **354.1** | **284.7** |
|  | **Cancers** | **206.0** | 180.1 | 232.0 | **207.1** | 169.1 | 245.1 | **163.9** | **148.1** |
|  | **CVD** | **306.2** | 270.1 | 342.4 | **282.4** | 236.8 | 327.9 | **304.5** | **247.8** |
|  | **CRD** | **14.0** | 9.0 | 19.0 | **12.9** | 5.5 | 20.3 | **16.9** | **11.1** |
|  | **DM** | **9.9** | 8.5 | 11.3 | **10.2** | 8.2 | 12.3 | **7.6** | **6.7** |
| **Russia** | **Total** | **427.7** | 270.3 | 585.1 | **311.0** | 102.8 | 519.1 | **560.5** | **442.8** |
|  | **Males** | **586.2** | 360.2 | 812.2 | **412.2** | 113.1 | 711.3 | **807.6** | **631.1** |
|  | **Females** | **317.8** | 237.4 | 398.2 | **261.4** | 163.2 | 359.5 | **349.0** | **281.4** |
|  | **Cancers** | **209.5** | 168.3 | 250.8 | **211.1** | 151.2 | 271.1 | **161.7** | **144.4** |
|  | **CVD** | **215.7** | 99.4 | 332.1 | **110.1** | -43.7 | 263.9 | **380.3** | **279.8** |
|  | **CRD** | **19.5** | 10.1 | 29.0 | **22.3** | 8.3 | 36.2 | **12.8** | **11.4** |
|  | **DM** | **14.4** | 11.7 | 17.0 | **16.9** | 13.1 | 20.7 | **5.7** | **7.2** |
| **Tajikistan** | **Total** | **490.8** | 451.8 | 529.9 | **505.9** | 454.8 | 557.0 | **325.3** | **305.7** |
|  | **Males** | **552.7** | 514.3 | 591.1 | **564.6** | 514.6 | 614.6 | **381.3** | **351.3** |
|  | **Females** | **425.0** | 389.0 | 460.9 | **439.8** | 393.2 | 486.4 | **270.0** | **260.8** |
|  | **Cancers** | **111.0** | 94.8 | 127.2 | **116.1** | 92.2 | 140.1 | **74.7** | **66.7** |
|  | **CVD** | **311.7** | 287.7 | 335.7 | **317.6** | 286.1 | 349.1 | **213.0** | **198.4** |
|  | **CRD** | **25.5** | 13.9 | 37.2 | **27.3** | 11.5 | 43.1 | **18.1** | **16.1** |
|  | **DM** | **45.5** | 41.0 | 49.9 | **51.1** | 45.4 | 56.7 | **19.5** | **24.5** |
| **Turkmenistan** | **Total** | **624.8** | 522.3 | 727.3 | **651.5** | 519.7 | 783.3 | **374.1** | **370.0** |
|  | **Males** | **735.2** | 618.3 | 852.1 | **766.7** | 604.0 | 929.4 | **460.4** | **452.5** |
|  | **Females** | **496.1** | 414.5 | 577.6 | **528.5** | 416.3 | 640.7 | **290.9** | **288.2** |
|  | **Cancers** | **130.7** | 115.3 | 146.1 | **140.0** | 118.2 | 161.8 | **73.8** | **75.9** |
|  | **CVD** | **431.2** | 366.3 | 496.2 | **446.1** | 355.7 | 536.4 | **273.6** | **266.3** |
|  | **CRD** | **8.8** | 2.4 | 15.1 | **9.9** | 0.9 | 18.9 | **6.8** | **5.7** |
|  | **DM** | **38.7** | 35.4 | 42.0 | **41.7** | 37.5 | 45.9 | **19.9** | **22.1** |
| **Uzbekistan** | **Total** | **634.4** | 572.1 | 696.7 | **654.3** | 567.0 | 741.6 | **417.7** | **394.0** |
|  | **Males** | **756.8** | 684.4 | 829.2 | **776.9** | 675.3 | 878.5 | **505.1** | **478.3** |
|  | **Females** | **517.7** | 461.9 | 573.4 | **536.7** | 458.7 | 614.7 | **335.2** | **314.6** |
|  | **Cancers** | **132.9** | 127.0 | 138.9 | **142.4** | 134.1 | 150.8 | **77.5** | **77.1** |
|  | **CVD** | **394.4** | 351.5 | 437.3 | **384.0** | 326.9 | 441.1 | **295.8** | **273.4** |
|  | **CRD** | **19.1** | 15.0 | 23.1 | **21.6** | 15.7 | 27.5 | **11.3** | **9.6** |
|  | **DM** | **58.0** | 54.2 | 61.8 | **61.9** | 56.8 | 67.0 | **33.1** | **33.9** |
| **Ukraine** | **Total** | **800.0** | 567.8 | 1032.1 | **807.0** | 477.8 | 1136.1 | **515.4** | **530.0** |
|  | **Males** | **1136.4** | 826.4 | 1446.3 | **1127.3** | 636.0 | 1618.6 | **719.3** | **782.6** |
|  | **Females** | **500.1** | 353.4 | 646.7 | **508.7** | 281.8 | 735.6 | **343.7** | **315.5** |
|  | **Cancers** | **254.5** | 199.3 | 309.6 | **259.7** | 172.7 | 346.6 | **158.2** | **166.8** |
|  | **CVD** | **519.5** | 373.2 | 665.8 | **523.6** | 292.6 | 754.6 | **340.0** | **347.9** |
|  | **CRD** | **25.1** | 14.9 | 35.2 | **31.9** | 16.6 | 47.2 | **12.4** | **10.6** |
|  | **DM** | **7.5** | 5.0 | 10.0 | **7.8** | 3.9 | 11.7 | **4.7** | **4.7** |
